# Validation of a Novel Boxing Monitoring System to Detect and Analyse the Centre of Pressure Movement on the Boxer’s Fist

**DOI:** 10.3390/s21248394

**Published:** 2021-12-16

**Authors:** Tobias Menzel, Wolfgang Potthast

**Affiliations:** Institute of Biomechanics and Orthopaedics, German Sport University Cologne, 50933 Cologne, Germany; potthast@dshs-koeln.de

**Keywords:** instrumented sport equipment, boxing monitoring system, smart wearable, centre of pressure, biomechanics analysis

## Abstract

The examination of force distribution and centre of pressure (CoP) displacement is a common method to analyse motion, load, and load distribution in biomechanical research. In contrast to gait analysis, the force progression in boxing punches is a new field of investigation. The centre of pressure displacement and distribution of forces on the surface of the fist during a boxing punch is of great interest and crucial to understanding the effect of the punch on the biological structures of the hand as well as the technical biomechanical aspects of the punching action. This paper presents a new method to display the CoP progression on the boxer’s fist Therefore, this study presents the validation of the developed novel boxing monitoring system in terms of CoP displacement. In addition, the CoP progression of different punching techniques in boxing is analysed on the athlete’s fist. The accuracy of the examination method of the CoP course was validated against the gold standard of a Kistler force plate. High correlations were detected between the developed sensor system and the force plate CoP with a Pearson correlation coefficient ranging from 0.93 to 0.97. The information obtained throughout the experimental study is of great importance in order to gain further knowledge into the technical execution of boxing punches as well as to provide a novel measuring method for determining CoP on the surface of the fist, to improve the understanding of the etiology of boxing-related hand injuries.

## 1. Introduction

The analysis of biomechanical punch data such as punch force, punch acceleration, fist speed, and punch time are of great importance for the verification, assessment, and evaluation of punching technique and effectiveness and have therefore been investigated in numerous studies [1,2,3,4,5,6,7,8,9]. The results of these studies illustrate the high forces (up to 4200 N) which occur in the hand and finger area during a boxing punch. Based on this, the existing literature shows that the hand and finger area, in addition to the head region, have an increased potential for injury due to the high forces exerted during a punch [10,11,12,13,14]. The most risk-prone regions of the hand for injury are the radial carpals, metacarpals, and phalanges [12,13]. Although it can be assumed, based on the intended point of impact at the second and third metacarpophalangeal joints, that the force generated and transmitted through these joints can lead to an overload of the biological structures and thus be mainly responsible for the injuries, as yet little is known about the cause of hand and finger injuries during a boxing punch.

Furthermore, the examination of the force distribution and centre of pressure (CoP) displacement on the contact surface of the fist can provide valuable information about the area of impact and the striking technique performed by the athlete.

The examination of the CoP displacement and distribution of forces on the surface of the fist during a boxing punch is therefore crucial to understanding the effect of the punch on the biological structures of the hand as well as the technical biomechanical aspects of the punching action.

The examination of the force distribution and CoP displacement under the foot surface is a common method of the examination in biomechanical gait and running analysis [15,16] and is also taken into account as a performance variable in the execution of athletic movements [17,18]. By contrast, the investigation of the force progression in martial arts, as represented by boxing, is a new field of investigation. An extensive literature review has revealed only one study which investigated the distribution of force on the boxer’s fist during a punch. This study, by Loosemore, Lightfoot, Meswania, and Beardsley [11], investigated the distribution and magnitude of pressure and load between the knuckles using low standard Fuji Film Pressurex^®^ films placed on the knuckles of the boxer’s fist. This paper [11] is the first and thus only analysis which has investigated a variation of the impact force distribution across the second, third, fourth, and fifth metacarpophalangeal joints. Despite the utility of its findings, the method used has limited relevance to the analysis of CoP. The low standard Fuji Film Pressurex^®^ method can only be used to investigate single punches, as the pressure film strips can only be used once and must be carefully removed and analysed afterwards. Furthermore, this method does not allow for the examination of the temporal CoP displacement but only the representation of the force distribution between the metacarpophalangeal joints.

This review shows the gap in the existing research on CoP distribution on the fist contact surface during a boxing punch. Consequently, this paper presents an analysis of the CoP distribution during boxing by use of a novel boxing monitoring system [19]. This newly developed sensor system was extended to be able to represent the point of force application and the CoP distribution on the boxer’s fist surface [19]. Therefore, the CoP progression measured with the sensor system was validated in a first examination against a Kistler force plate, which is considered as the gold standard [20,21,22], before the viability of CoP analysis was assessed through real punch tests with the help of an experienced athlete. For this purpose, the four main punching techniques of the cross, jab, hook, and uppercut were performed.

The information obtained through the experimental study has great importance in laying the foundations for further investigation of the technical execution of boxing punches, providing a method to improve the understanding of the etiology of boxing-related hand injuries.

## 2. Materials and Methods

**Experimental Setup and Protocol**

**Figure 1:**
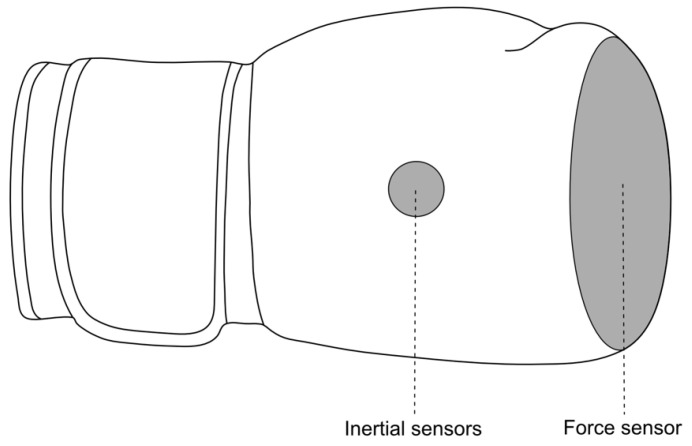
Schematic of the developed sensor system instrumented to the sport equipment [23].

To investigate the CoP movement, an innovative sensor system was used that was developed and validated in prior work [19]. The developed sensor system for measuring the CoP movement consists of force-sensing resistors that are based on the piezoresistive principle. The sensor calibration was conducted by gradient step impact tests using a Zwick/Roell material testing device, the testXpert^®^ III Version 1.4 (ZwickRoell GmbH & Co. KG, Ulm, Germany) as well as a Kistler Force plate using Vicon Nexus software. The gradient step tests were performed from 0 up to 2500 N with gradient steps of 50 N. The sensor-derived force is based on a calibration function using a polynomial function over the sensor-derived conductance as the sensor is measuring the relative change in electrical resistance by an applied pressure to the sensors through a reference resistor and a 3.3 V sensor input. The sport equipment itself is defined by its size and weight as well as the materials used and is an integral part of the official competition regulations. To instrument the boxing glove, the sensor system was therefore developed by the use of microtechnology and the development of customised flexible system components. A 4 × 4 sensor matrix runs along each of the individual anatomical structures of the digits in order to determine the CoP movement on the fist surface. The overall covered sensing area is thereby 106 × 106 mm of the glove’s punching area (Figure 1). In order to analyse the impact force, the sensor system was comprehensively validated in a previous study [19]. The conducted validation experiments demonstrated a significant accuracy for the determination of the impact force of R^2^ = 0.97 to R^2^ = 0.99 compared to a force plate [19]. The development of the sensors allowed the system to be developed with a maximum hysteresis of 1.91% and a 99.99% reduction in sensor creep after 0.28 s.

In the first part of the experimental study, a novel data collection method was validated as a means of detecting and analysing the CoP movement in various punching techniques in boxing. In this context, the focus was to validate the CoP movement of the developed sensor system across the x- and y-axis on the surface of the punching fist. The validation of the CoP movement was performed using a force plate, the gold standard in biomechanics [22], to validate the developed sensor system against a reference system.

In this validation, different boxing punches were applied with a specially-equipped boxing glove to the centre of a force plate. The developed sensor system was installed in a 12-ounce (340.194 g) AIBA certified boxing glove (2017 model) from Adidas (Adidas AG, Herzogenaurach, Germany). The data acquisition using the force plates was performed at a measuring frequency of 10,000 Hz and a measuring frequency of 1000 Hz for the developed sensor system in both the validation as well as punching experiment. The sensor-derived data were stored in a buffer to allow a comprehensive post processing and analysis using MATLAB. The measurement data obtained from the Kistler force plate were recorded using Vicon Nexus software for motion capture in life sciences.

**Figure 2:**
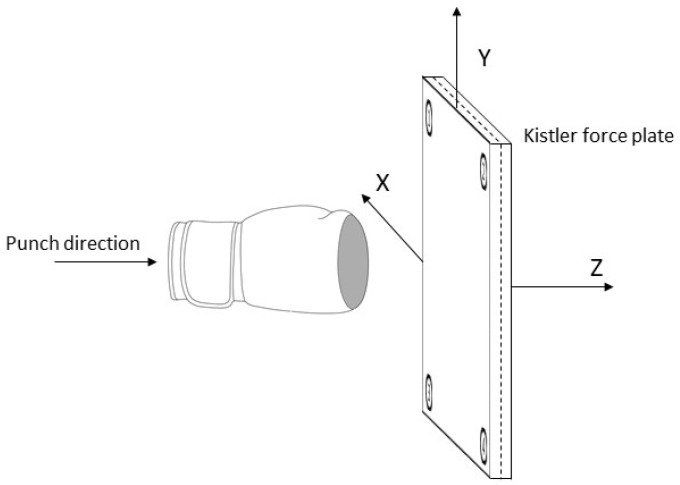
Test setup schematic [19].

Due to the gap of the existing literature, no accepted and generally valid research method or protocol for the validation of the force distribution during a boxing punch yet exists, a limitation also emphasised by Loosemore et al. [11]. Consequently, a customised test report was developed to validate the CoP, determined by the sensor in comparison with the force plate. For the validation of the CoP movement on the fist surface, a total of 25 blows from 500 up to 1800 N were applied to the force plate. The average contact time of the boxing glove with the force plate was 25.5 ms. The experimental protocol consisted of five validation runs. For each validation run, five blows were applied to the force plate for the analysis of CoP movement validation against the force plate (Figure 2). The blows were applied in a frontal direction onto the centre of the force plate to ensure that the sensing area was activated and to validate the sensor-derived CoP movement against the force plate-derived CoP movement. 

In the second part of the study, a total of 180 boxing strokes were performed with the help of an experienced athlete on a punching bag. During the experiment, the four main punching techniques of the jab, cross, uppercut, and rear hand hook were tested. The experiment protocol consisted of three test cycles with fifteen punch repetitions each. This protocol was carried out for each of the four striking techniques consecutively.

**Data Analysis**

For the comparison of the acquired CoP course data of the developed sensor system with the force plate, the measured sensor data had to be interpolated for validation purposes. Data interpolation was performed for a holistic analysis of the CoP course, due to the different data acquisition frequencies of the force plate (10,000 Hz) and the developed sensor system (1000 Hz). The collected biomechanical performance data, including the CoP course, were processed for further data analysis using custom-built MATLAB (2018b) (The MathWorks, Natick, MA, USA) routines. 

In order to ensure uniform data analysis of the first part of the sensor validation test, the CoP data obtained from the force plate and the new sensor system were normalized in order to start the centre of pressures at 0x and 0y position within the coordinate system. This ensured that any deviation between the two CoP profiles was clearly evident for validation purposes. For the subsequent experimental investigation of the CoP progression of the four punching techniques, the data were not normalised in this fashion. The centre of pressure course is presented in the form of 3D bubble charts. This form of visual representation enables the course of the centre of pressure in the x- and y-direction to be displayed in the coordinate system with the effective force at the instant in time, expressed in terms of the size of the individual bubbles.

**Figure 3:**
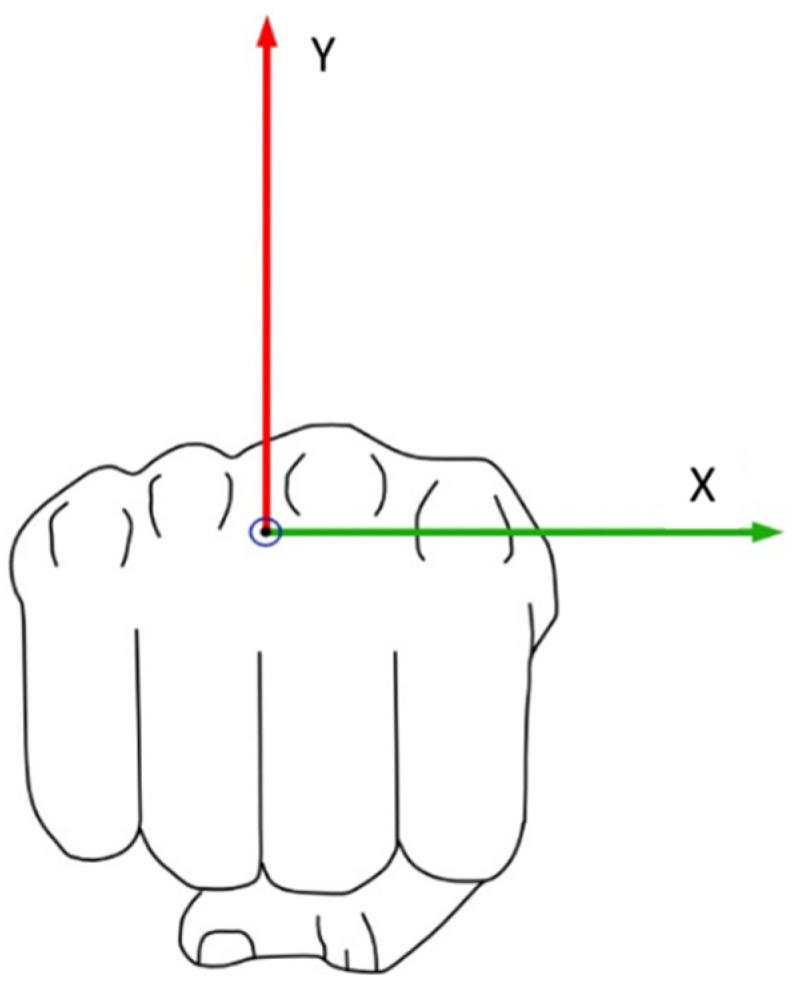
Frontal view of the right punching fist with highlighted metacarpal heads.

The graphical representation of the CoP progression is presented from a frontal view of the punching fist (the athlete’s right hand), as illustrated by Figure 3. For the strokes performed, the representation and orientation of the coordinate system of the centre of pressure progression during the cross, hook, and uppercut is to be understood from the frontal view of the athlete’s left hand (Figure 3), whereas the centre of pressure progression for the jab stroke technique is to be understood from the athlete’s right hand.

**Assessment Variables and Statistical Analysis**

The first step in analysing the data sets was performed by comparing the mean values and standard deviation. This step served to analyse the data sets in terms of correlation analysis in order to evaluate the magnitude of concordance between the data sets. For the overall analysis, the mean and standard deviation of the test cycles were calculated and reported. A Bland–Altman analysis was applied for the graphical comparison of the displacement in the x- and y-direction as well as punch force for the force plate measurement method with the developed sensor system. Additionally, the root mean square error for the centre of pressure progression in the x- and y-direction was calculated for further comparison. The generated CoP courses of the force plate and sensor system were compared for the validation method using the Pearson correlation coefficient. The statistical data analysis was conducted using the IBM SPSS Statistics software for Windows, version 23.0 (IBM Corporation, New York, NY, USA).

**Participants**

The primary focus of this scientific experimental investigation was to determine the possibility of measuring and analysing the CoP displacement during a boxing punch using the developed sensor system. The first part of the study focused on examining the validity of the sensor system against the gold standard of a force plate as the reference system. In the second step, a boxing athlete with more than 10 years of boxing experience was included to test the method in a practical environment, to generate the CoP course data on the boxer’s fist while punching. The subject was briefed in advance of the experiment on data collection and the experimental protocol. In order to avoid technical misinterpretation regarding the execution of the punches, the subject was informed in detail about the four punching techniques to be executed based on the technique model of the cross, jab, uppercut, and hook. These instructions were used to prevent the execution of incorrect punching techniques and to ensure the reproducibility of the punches to be tested. In order to follow the technique model of a realistic punch, the subject was instructed to perform the punching technique as if in a competition. This included the fast execution of the stroke as well as the immediate return to the defensive position. This was to avoid the execution of punches that go beyond the realistic punching technique in sparring or competition in order to analyse and detect the boxing specific CoP progression. After the execution of the blow, the subject was therefore instructed to immediately return to the defensive position. In addition, the test person was informed about the risks and benefits of the experiment. The measurements were conducted in the biomechanical laboratory of the German Sport University Cologne. Prior to the experimental testing, the subject undertook a boxing-specific warm up for muscle activation and to become familiar with the experimental setting and the equipment to be utilised for data acquisition.

## 3. Results

**Figure 4:**
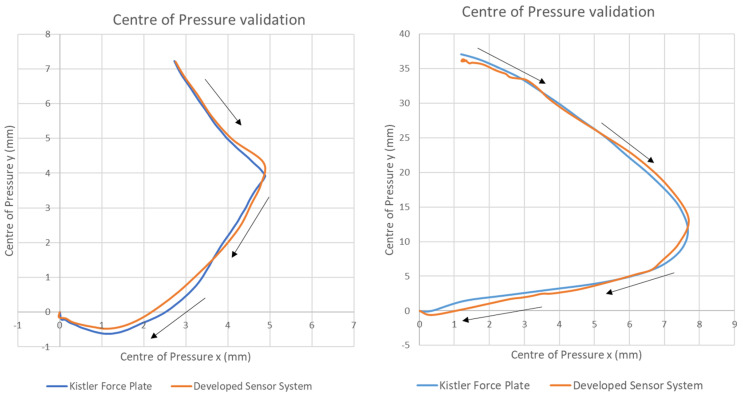
Centre of pressure validation force plate vs. developed sensor system example test runs.

**Figure 5:**
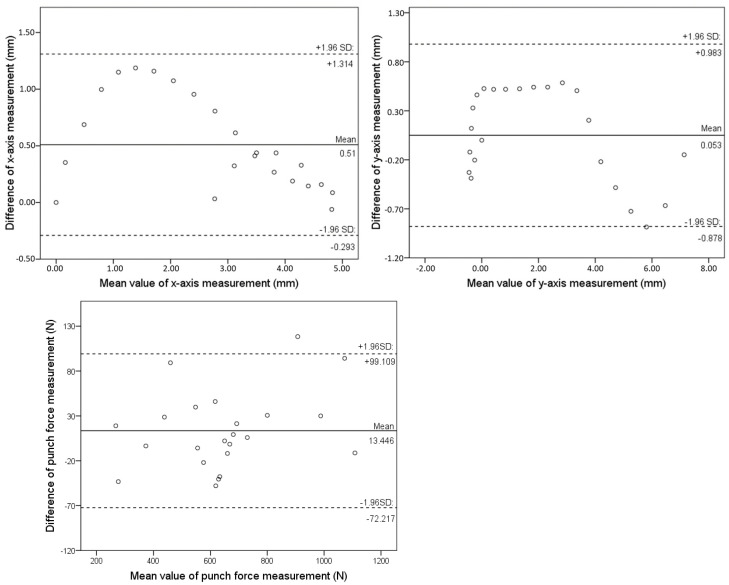
Bland–Altman analysis for force plate vs. developed sensor system example test run.

The accuracy of the developed boxing monitoring system for measuring the CoP course was analysed by comparing the new sensor system against the gold standard Kistler force plate. To present the validation results, two example impact tests are shown in Figure 4. These figures show the sensor-derived CoP distribution visually compared to the force plate-derived CoP distribution across the x- and y-axes. This comparison of both systems shows a high degree of agreement. Additionally, Table 1 presents the Pearson correlation coefficient. The Pearson correlation coefficient ranged from 0.93 to 0.97 in the x-direction and from 0.97 to 0.99 in the y-direction. This corresponds to an average Pearson correlation coefficient of 0.96 (SD = 0.03) on the x-axis and an average Pearson correlation coefficient of 0.98 (SD = 0.01) on the y-axis. The quality of the applied calibration routines was also examined and evaluated by means of the root mean square error (RMSE). The results presented in Table 1 demonstrate that an average root mean square error of 0.87 mm to 3.13 mm was determined in the x-direction. A larger deviation was found in the CoP along the y-axis with an average RMSE of 0.51 mm to 4.19 mm for the impact tests performed against the force plate. This corresponds to a RMSE percentage of 0.82–2.95% in the x-axis and 0.48–3.95% in the y-axis relative to the sensor area. The overall results of the validation study show an average RMSE of 1.62 mm (SD = 1.30 mm) in the x-direction and an average of 1.83 mm (SD = 2.05 mm) in the y-direction. The results of the Bland–Altman analysis were used to evaluate the bias between the mean differences of the developed sensor system compared to the gold standard of the force plate. The results show that 95% of the developed sensor system differences compared to the force plate lay within the statistical limits as presented in Figure 5 for the CoP in the x- and y-direction as well as the punch force.

**Figure 6:**
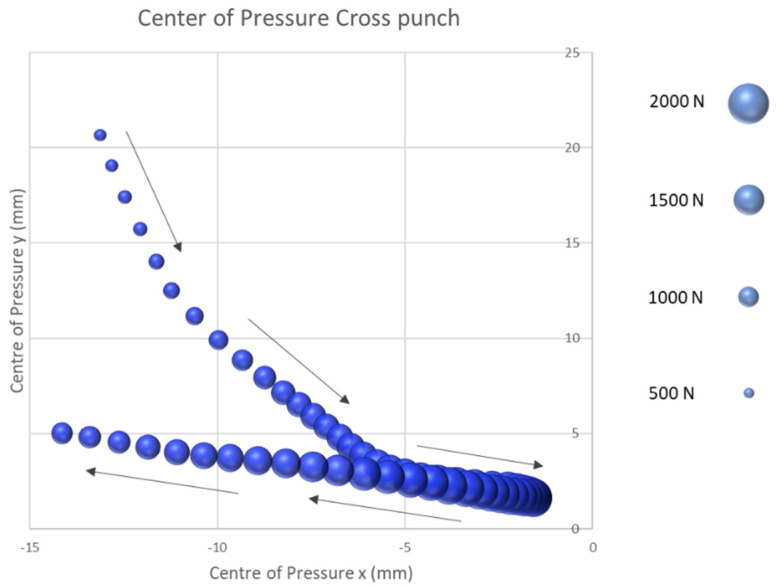
Centre of pressure cross punch.

Based on the validation results, the study continued to investigate the CoP course on the surface of the boxer’s fist during a boxing punch. The first technique tested was the straight cross. Figure 6 shows the average trajectory of the CoP on the surface of the fist when the fist hit the target with the effective force at the instant in time illustrated by the size of the individual bubbles. The trajectory shows the start of the CoP movement between the second and third metacarpophalangeal joints. From the second and third metacarpophalangeal joints, the centre of pressure is moving in a triangular pattern around −19.03 mm on the x-plane and 11.54 mm on the y-plane in a distal medial direction to the third proximal phalanges. At this point, the impact reaches the maximum impact force of 1753.4 N (SD = 485.92 N) on average for the subject tested. After reaching the maximum impact force the centre of pressure moves 12.56 mm in the x-direction and 3.4 mm in the y-direction, lateral to the second proximal phalanges. At this point, the fist separates from the target and is returned to the defensive position.

**Figure 7:**
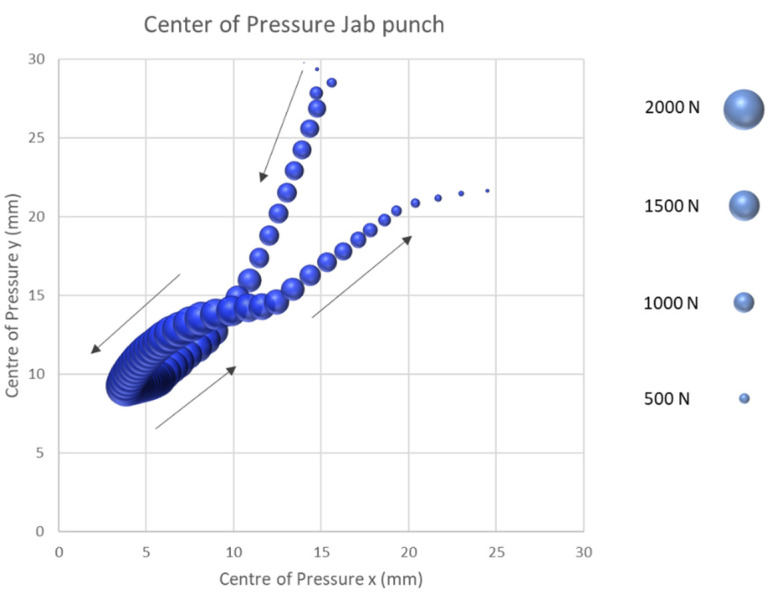
Centre of pressure jab punch.

The second straight boxing punch technique tested was the contralateral jab. Similar to the cross, the centre of pressure course for the jab technique starts between the second and third metacarpophalangeal joints (Figure 7). In a triangular course, the centre of pressure leads from the second and third metacarpophalangeal joint by −20.77 mm on the x-axis and −20.63 mm on the y-axis in the direction of the third proximal phalanges. With an average punch force of 973.85 (SD = 542.83 N), the stroke reaches its maximum strike force at the fist’s anatomical position on the striking hand. After the maximum impact force has been obtained, the centre of pressure moves 20.76 mm in the medial and 12.48 mm in the proximal orientation in the direction of the second proximal phalanges. From the second proximal phalanges, the fist separates from the object to be hit in order to resume to the defensive position and execute a new strike.

**Figure 8:**
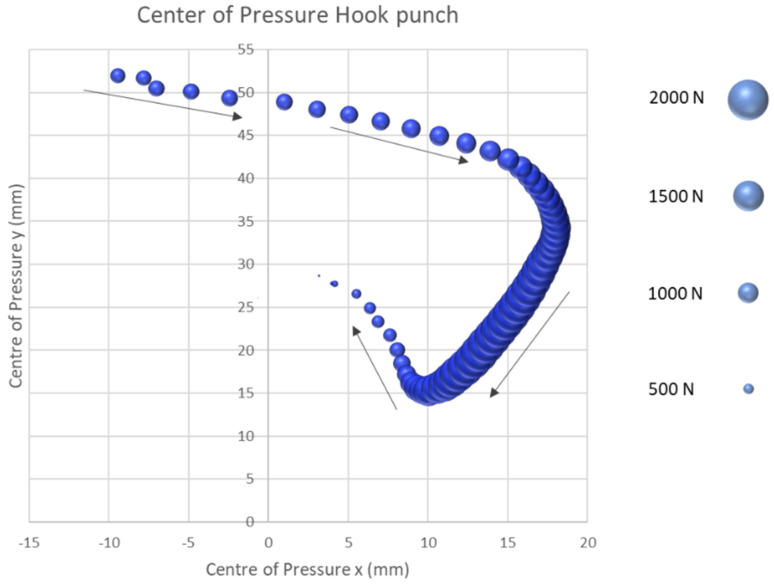
Centre of pressure hook punch.

After the successful execution of the two straight punching techniques, the study investigated the first semi-circular punching technique of the rear hand hook. The beginning of the rear hand hook shows a start of the CoP course on the third metacarpophalangeal joint, as presented in Figure 8. From the third metacarpophalangeal joint, the CoP extends 15.04 mm on the x-axis and 9.74 mm on the y-axis in the direction of the fifth proximal phalanges. Without reaching the maximum impact force, the CoP leads in the medial direction by −5.0 mm on the x-coordinate and −22.53 mm on the y-coordinate in the direction of the fourth proximal phalanges. At this point, the hook reaches the mean maximum impact force of 1407.39 N (SD = 168.27 N). Subsequently, the CoP progresses by an average of 2.63 mm in the medial and 4.08 mm in the distal direction. The fist detaches from the target after the CoP has progressed by 6.41 mm in the medial and 12.16 mm in the proximal direction at the fourth proximal phalanges (Figure 8).

**Figure 9:**
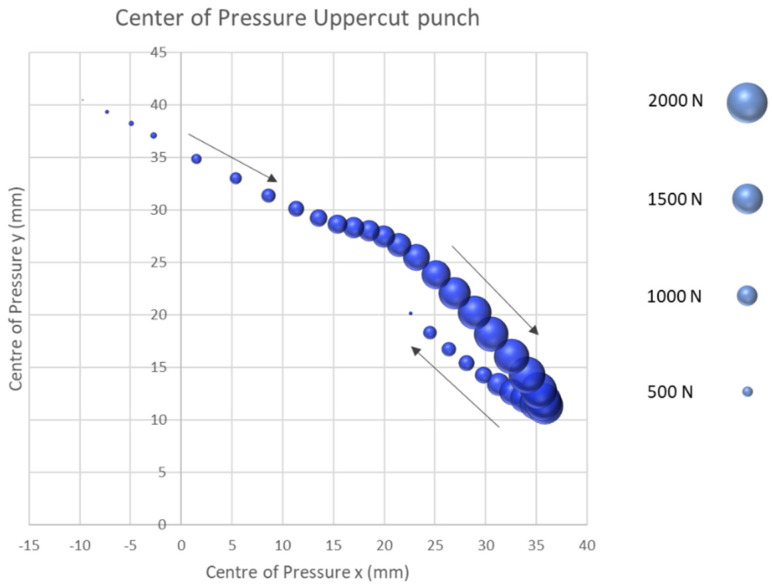
Centre of pressure uppercut punch.

The uppercut was the last tested punching technique. As shown in Figure 9, the CoP starts at the third proximal phalanges. In a distal lateral movement, the CoP is moving 47.6 mm in the x-direction and 30.64 mm on the y-axis, in the direction of the fifth proximal phalanges. At this anatomical position, the uppercut exhibits the subject’s mean maximum impact force of 1397.38 N (SD = 276.88 N). After the maximum impact force is obtained, the CoP moves on average −13.2 mm in the medial and 8.82 mm in the proximal direction to the fourth proximal phalanges. The fist is released from the target at this point and is returned to the subject’s defensive position.

## 4. Discussion

To the author’s knowledge, this is the first study that has analysed the CoP course on the boxer’s fist. Therefore, it is the first time that a differentiation has been made with respect to the course of the CoP on a boxer’s fist between the four main punching techniques of the jab, cross, rear hand hook, and uppercut.

The investigation carried out in the first part of this paper, validating the CoP progression using a newly developed sensor system, demonstrated good results when compared with a force plate. The statistical analysis of the presented CoP courses was performed using the Pearson correlation coefficient, the RMSE, and the Bland–Altman analysis. The results, shown in Table 1, demonstrate a high correlation between the measurements of the developed sensor system and the force plate as well as a low RMSE relative to the sensor area (>4.0 %). The calculated Pearson correlation coefficient ranges from 0.93 to 0.97 on the x-axis with a mean of 0.96 (SD = 0.03). The validated y-axis determination of the CoP course showed a higher correlation, ranging from 0.97 to 0.99, with a mean of 0.98 (SD = 0.01). The calculated RMSE showed an error range of 0.87 mm to 3.13 mm for the CoP on the x-axis. By contrast, the y-axis displayed an error range from 0.51 mm to 4.19 mm. With all validation cycles performed, the overall RMSE was 1.62 mm (SD = 1.30 mm) on the x-axis and 1.83 mm (SD = 2.05 mm) on the y-axis. No statistically significant difference was analysed between the RMSE in the x- and y-direction. Possible causes for the deviation in the x- and y-axis of the CoP curve and the RMSE are, for instance, the positioning of the sensors on the boxer’s fists and the particular size of the individual sensors within the matrix. The sensor positioning in the glove was designed and developed in order to cover the entire potential impact surface. Although the official contact area can be covered by the sensor system, hits with the side of the glove or the open glove, for example, which do not comply with the official rules, can lead to a displacement of the contact area and thus of the CoP. The size of the individual sensors has been minimised and positioned in order to analyse crucial areas of the fist anatomy, such as the metacarpophalangeal joints and proximal phalanges, with a maximum number of sensors. Since the CoP is calculated with the help of the sensors starting from the sensor’s centre, slight deviations may occur due to the calculation method and thus the course of the CoP. These limitations have been reduced to a minimum due to the sensor design and developed, providing excellent accuracy in reproducing the CoP on the surface of the fist during a boxing punch compared to a force plate. It has to be considered that even the gold standard of a force plate has its limitations [24,25]. The error of a force plate is not zero. Studies have shown that especially when the normal forces perpendicular to the plates surface are small compared to the horizontal forces and/or when the force application point is located at the edge of the force plate, the force plate error will increase. An error can be caused by the different impact techniques in the second part of the study, since different horizontal forces are generated in relation to the normal forces, which could lead to errors. Especially at the very beginning and the end of the contact phase, these errors can occur, since at these times the forces are relatively small. To reduce these errors, special care was taken in the execution of the impact on the force plate [24,25].

The results of the second part of the study demonstrated that the start of the CoP in all four punching techniques tested started between the second and third metacarpophalangeal joint. By comparison, the two semi-circular impact techniques of the rear hand hook and the uppercut show a shift of the start of the CoP by about 5.0 mm in the medial direction and by 22.5 mm in the proximal direction. This shift can be explained by the stronger diffraction in the transversal axis of the fist at the moment of impact compared to the jab or cross and can be understood as the first technical optimization approach.

Following the start of the force application, the CoP in all punches proceeds in a distal lateral direction to the centre of the striking fist. For the two straight punching techniques, the cross and the jab, the maximum punching force is reached in the centre of the fist. By contrast, the rear hand hook and uppercut punching techniques exhibit a CoP which continues beyond the centre in a lateral direction towards the fourth and fifth proximal phalanges until the maximum punching force is obtained. After the maximum force has been reached, the centre of pressure proceeds in a medially proximal orientation back towards the second and third metacarpophalangeal joint for all punches tested. For the rear hand hook and uppercut techniques, the CoP ends at the height of the third proximal phalangeal joint. For the two straight punching techniques, the CoP ends at the level of the second proximal phalanges. Subsequently, the fist is released from the target and returned to the defensive position. This investigation showed unique displacement patterns in the different punching techniques.

The results of this study suggest that the developed sensor system provides a novel method for determining the CoP on the surface of the fist. Furthermore, the results were able to represent a specific course of the CoP of the four tested types of boxing punches on the surface of the fist. These results could therefore be a further means for evaluating the striking technique in order to assess the CoP of the second and third metacarpal according to Arus [26] for optimal force transmission. The results of this study cannot only be used for a technical analysis but can also provide incisive insights into the detection and prevention of hand and finger injuries. As described above, the subject in this study showed a strongly laterally aligned CoP course and a maximum impact force at approximately the fourth and fifth proximal phalanges of the two semi-circular impact techniques, which exposes the anatomical structures to a significantly higher load than compared to the straight punching techniques of the cross and jab punch. A more detailed examination of the injury history of the subjects could be of great importance in follow-up studies to determine whether injuries have occurred in these hand areas in the past.

Since the research field of the CoP and the force distribution on the surface of the fist during a punch is a novel one, there are no existing findings which show a connection between the force of the punch and the force distribution with resulting injuries. Additionally, there are currently no scientific studies investigating risk factors which predominate for hand and finger injuries. Based on the current state of research, it is thus only possible to make cautious inferences regarding the factors that may lead to these types of injuries [11]. Potential influencing factors include the magnitude of the impact force, the striking technique used, and thus the distribution of the force, as well as the CoP progression on the fist and the degree of fist clench at the time of impact of the fist on the targeting object.

Further studies are necessary to investigate the relationship between impact techniques, impact forces, the CoP distribution, and possible injury risks. For this purpose, a larger number of test persons with different levels of experience should be tested to investigate this issue in depth. The analysis of variation between experienced and inexperienced test persons has already shown significant differences [3,4,7,19]. Such investigations of different levels of experience are crucial, as Zazryn et al. [27] have demonstrated that more injuries occur in amateur boxing than in professional boxing. Currently, no scientific literature provides information about the causes of this difference in injury frequency between experienced and inexperienced subjects. A study of different levels of experience is therefore critical for understanding punching force and punching technique as a variable affecting the frequency of hand and finger injuries. In addition, the injury history of the participating subjects should be included in follow-up studies. Consequently, a possible connection with the executed punch technique and the overstrain of anatomical structures of the hand would be possible. Investigation during real competition and sparring matches would be an additional enlightening extension of the current study, since, as shown in the results of the study by Porter and O’Brien [28], as well as by Zazryn, Cameron, and McCroy [27], the frequency of injuries was significantly higher during competition than during training. Thus, the results of a follow-up study could provide information on a change in punching technique and/or punch force during competition and training situations that are associated with potential hand injuries.

A significant limitation of the current study is the investigation of the CoP as a technique constant in single executed punches. Davis and Wittekind [29] have shown in their study that punch combinations account for a large proportion of the punches performed during a competition. In order to further investigate the CoP during a boxing punch and the punching technique performed, follow-up studies should therefore focus not only on single punches but also on punch combinations and the course of the CoP between punches within a combination frequency. Furthermore, it should be noted that the punches were made against a punching bag. The course of the CoP cannot therefore be assimilated to a punch against a head. In order to investigate the CoP with a blow to the head, sparring or competition would have to be analysed as described above. Alternatively, a head imitation could be used for further studies. 

The subject investigated in this experimental study did not use hand bandages during the tests. Prusak et al. [30] have shown that special taping techniques cause a change in the CoP course on the foot and thus limit the risk of injuries. It is therefore essential to further investigate to what extent the use of tape or hand bandages and the applied technique causes a change in the CoP course and thus could provide preventive protection against excessive strain on anatomical areas due to the punching technique performed.

## 5. Conclusions

The main purpose of this paper was to display the sensor system’s potential for detecting and presenting the course of the CoP in the four tested boxing punching techniques. The results demonstrate that the newly developed boxing monitoring system enables the examination and display of the centre of pressure on the surface of the fist during a boxing punch with great accuracy of up to R 0.99 when compared to the gold standard force plate. The study has not only shown the viability of the method to use piezo-resistive pressure sensors for CoP determination, but has also provided new insights into CoP progression during various boxing techniques of the cross, jab, uppercut, and hook. Consequently, this paper shows that the punching technique has a decisive influence on the change of the CoP on the surface of the fist, as well as how the acting force evolves throughout the contact period and thus to the biological structures of the striking fist. The results of the study showed that the CoP progression is strongly dependent on the impact technique performed and that these techniques contain a unique individual repeatable CoP progression for the tested experienced athlete. The results indicate that the two straight punching techniques show a triangular force progression between the second and third metacarpophalangeal joint while the two semi-circular punching techniques display a CoP progression that extends to the fourth and fifth metacarpophalangeal joint, respectively, in the tested athlete. These results also reveal the area that is exposed most during a boxing punch and how long the biological structure is exposed to the acting force. 

The information obtained based on the presented method of force progression representation is fundamental for applying and conducting future field studies to expand the scientific understanding in terms of biological loaded structures in the sport of boxing. Further studies, building on this preliminary work, are necessary to investigate the potential link between changing CoP during a punch and hand and finger injuries. The results of the study can also be used in a future application as a performance monitoring tool. With such a monitoring solution, coaches and athletes can perform an in-depth technique analysis in order to optimise the striking technique and efficiency while reducing the potential risk of injury at the same time.

## Figures and Tables

**Table 1:** Pearson correlation coefficient and root mean square error for centre of pressure validation.

	Pearson Correlation Coefficient x	Pearson Correlation Coefficient y	RMSE x (mm)	RMSE y (mm)
Min	0.93	0.97	0.87	0.51
Max	0.97	0.99	3.13	4.19
Mean ± Std.	0.96 ± 0.03	0.98 ± 0.01	1.62 ± 1.30	1.83 ± 2.05

## Data Availability

The data presented in this study are available on request from the corresponding author. The data are not publicly available due to further research work. Data will be made available in the near future.
